# Genome‐Wide 5‐Methylcytosine and 5‐Hydroxymethylcytosine Signatures Analysis of Plasma Cell‐Free DNA in Schizophrenia

**DOI:** 10.1002/mco2.70293

**Published:** 2025-07-30

**Authors:** Gang Xue, Xia Wei, Li Li, Qi Zhang, Shanming Liu, Jun Zhang, Wen Hu, Qiannan Zhao, Wenjing Zhang, Chunyan Luo, Qiyong Gong, Bo Zhang, Dan Xie, Su Lui

**Affiliations:** ^1^ Laboratory of Omics Technology and Bioinformatics Frontiers Science Center for Disease‐related Molecular Network State Key Laboratory of Biotherapy West China Hospital Sichuan University Chengdu Sichuan China; ^2^ Department of Radiology and Functional and Molecular Imaging Key Laboratory of Sichuan Province West China Hospital of Sichuan University Chengdu China; ^3^ Research Unit of Psychoradiology Chinese Academy of Medical Sciences Chengdu China; ^4^ Department of Nuclear Medicine West China Hospital Sichuan University Chengdu China; ^5^ Mental Health Center West China Hospital Sichuan University Chengdu China; ^6^ Tailai Inc. Chengdu Sichuan China

**Keywords:** schizophrenia, cell‐free DNA, 5‐methylcytosine, 5‐hydroxymethylcytosine, magnetic resonance imaging

## Abstract

Schizophrenia (SCZ) is a highly heritable neuropsychiatric disorder that affects ∼1% of people globally. Despite extensive research, there remains a lack of biomarkers for SCZ diagnosis and disease pathogenesis delineation. Cell‐free DNA (cfDNA), which carries the genetic and epigenetic signatures of origin tissue cells, may provide a noninvasive method for biomarker discovery. We performed cfDNA 5‐methylcytosine (5mC) and 5‐hydroxymethylcytosine (5hmC) sequencing of plasma samples from 66 individuals with SCZ and 77 healthy controls. We identified 954 differentially 5mC methylated regions (DMRs) and 1474 differentially 5hmC hydroxymethylated regions (DhMRs) that showed distinct patterns between SCZ and control samples. Many DMRs and DhMRs were associated with genes specifically expressed in brain tissues and were enriched in neuronal functions, as well as were enriched for genome‐wide association study (GWAS) of psychiatric and brain volume traits. Additionally, colocalization analysis revealed that DhMRs but not DMRs locations significantly overlapped with GWAS‐identified genomic loci of SCZ. Moreover, we observed associations between DMRs and DhMRs with brain regional measurements depicted by magnetic resonance imaging. Together, our findings indicated that cfDNA 5mC and 5hmC patterns are accessible epigenomic signatures that can serve as potential biomarkers and to help delineate SCZ pathogenesis.

## Graphical abstract

We performed cfDNA 5‐methylcytosine (5mC) and 5‐hydroxymethylcytosine (5hmC) sequencing of plasma samples from 66 individuals with SCZ and 77 healthy controls (HC). We identified differentially 5mC methylated regions (DMRs) and differentially 5hmC hydroxymethylated regions (DhMRs) that showed distinct patterns between SCZ and control samples. The analysis of DMRs and DhMRs as well as brain structure provided multiple insights into SCZ pathogenesis.

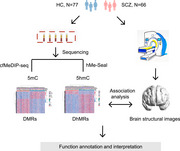

## 1 Introduction

As one of the main severe mental disorders, schizophrenia (SCZ) affects ∼1% of the global population [1]. However, a lack of objective biomarkers remains the challenge for SCZ diagnosis and investigating SCZ pathogenesis and leads to poor clinical performance in the treatment of individuals with SCZ [2]. Understanding the pathogenesis of SCZ is one of the 125 key questions for human beings [3]. Genome‐wide association studies (GWASs) have identified a growing number of significant loci associated with SCZ. However, only a few of these loci have been functionally characterized [4, 5, 6], and the high frequency of these loci in the general population precludes their clinical use.

Both genetic and environmental factors influence SCZ development [7]. Epigenomic studies can enhance our understanding of the underlying mechanisms and aid in biomarker discovery. 5‐Methylcytosine (5mC) DNA methylation is the major regulator of gene expression and is considered to represent the interplay between genetic and environmental factors [8]. Recent studies in brain tissue have revealed a correlation between alterations in 5mC and the occurrence of SCZ [9, 10]. In addition, genome‐wide methylation quantitative trait loci analysis indicated that SCZ GWAS‐associated single‐nucleotide polymorphisms (SNPs) influence the epigenetic plasticity of the brain [11]. Moreover, 5mC can be converted to 5‐hydroxymethylcytosine (5hmC) by ten‐eleven translocation (TET) family dioxygenases [12]. 5hmC modifications are most highly abundant in brain tissue, where they are present in up to 10‐fold greater levels than in other tissues [13]. Aberrant 5hmC has been associated with the mechanisms underlying many neurological diseases, including autism, Rett syndrome, and SCZ [14, 15, 16]. However, the acquisition of brain tissue is extremely challenging and does not reflect the state of the brain in vivo. In contrast, peripheral blood is more easily accessible, and the consistent DNA methylation signatures between the brain and peripheral blood tissue were observed [17]. In addition, changes in the blood DNA methylome in individuals with SCZ have shown potential for identifying diagnostic biomarkers [18]. Hence, peripheral blood may serve as a valuable surrogate for brain tissue for investigating the roles of 5mC or 5hmC in SCZ development.

Compare with peripheral blood DNA, there are some advantages of cell‐free DNA (cfDNA) in plasma in the detection of SCZ and the interpretation of its underlying mechanisms. First, unlike methylation patterns in only peripheral blood cells [18], cfDNA bearing the genetic and epigenetic signatures of origin tissue cells [19] may provide more information about the biological processes in SCZ brain tissues. The primary contributors to cfDNA are of hematopoietic origin, including granulocytes, erythrocyte progenitors, monocytes, and lymphocytes [20]. As a result, cfDNA inherently contains DNA derived from peripheral blood. In addition, cfDNA also encompasses DNA from solid tissue sources [20]. Notably, Jiang and colleagues [21] observed that cfDNA levels in individuals with SCZ were greater than those in matched healthy controls (HCs), and they proposed that the cfDNA in plasma was possibly derived from immunocytes and neural cells. Similarly, Lubotzky and colleagues [22] determined that the concentrations of total cfDNA and brain‐derived cfDNA were both greater in individuals with SCZ than in HCs. The brain‐derived cfDNA methylation markers can distinguish in individuals with SCZ from HCs [22]. Second, the half‐life of cfDNA is notably short, ranging from 5 to 150 min [23]. This characteristic facilitates real‐time monitoring of SCZ progression and treatment responses, offering a dynamic perspective on the disease. Last, the extraction process for cfDNA is simpler and more efficient compared with that of DNA from peripheral blood, enhancing its feasibility for clinical applications. However, genome‐wide epigenetic modification profiling of cfDNA from individuals with SCZ is still lacking. Advances in sequencing techniques, such as cell‐free methylated DNA immunoprecipitation and high‐throughput sequencing (cfMeDIP‐seq) [24], as well as cell‐free 5hmC sequencing based on selective chemical labeling (hMe‐Seal) [25], provide the opportunity to study whole‐genome cell‐free 5mC and 5hmC modifications, respectively, in individuals with SCZ.

Structural magnetic resonance imaging (sMRI), which is noninvasive, involves nonionizing radiation, has high soft tissue resolution and discrimination, and provides morphological information for human brain imaging in vivo. Numerous sMRI studies in individuals with SCZ have identified brain structural alterations in, affecting both cortical regions [26, 27] and subcortical nuclei [28, 29]. Interaction between genetics and the environment is considered to influence brain structure in individuals with SCZ [30]. The whole‐brain volume intrapair similarity was observed to increase with closer genetic relationships [31]. Moreover, consistent findings in sMRI studies have revealed brain anatomical differences within monozygotic pairs discordant for SCZ [31, 32], which indicates that the cause of SCZ is not entirely genetic. Indeed, DNA methylation is related to brain volume changes in individuals with SCZ [33]. Therefore, investigating the relationships between epigenetics and brain structure via sMRI might provide new information for understanding SCZ pathogenesis.

On this basis, we proposed three primary questions: (1) whether alterations in epigenetic modifications of cfDNA serve as potential objective signatures for SCZ; (2) whether these alterations can provide insights into SCZ pathogenesis; and (3) whether these alterations are associated with changes in brain structure. To address these questions, we first identified the different cell‐free 5mC and 5hmC modifications between individuals with SCZ and HC. Furthermore, we annotated the different epigenetic modifications to genes and then performed tissue and Gene Ontology (GO) enrichment analysis. Moreover, we conducted GWAS enrichment analysis and colocalization analysis to investigate whether different epigenetic modifications are associated with genetic variations linked to SCZ. Finally, we correlated different epigenetic modifications with brain structures measured by sMRI. We expect this study to provide a new strategy for identifying objective signatures for SCZ and for exploring the role of epigenetic modifications in SCZ development.

## 2 Results

### 2.1 Study Participants

In this study, we included 66 SCZ patients and 77 matched HCs (Table 1), with no significant intergroup differences in age or gender. Cognitive function and clinical symptom severity were assessed in all participants using the Brief Assessment of Cognition in Schizophrenia Scale (BACS) and the Positive and Negative Symptom Scale (PANSS), respectively. Compared with HCs, SCZ patients exhibited significantly impaired cognition (*p* value <0.001) and elevated psychiatric symptom severity (*p* value <0.001). Detailed subject information is shown in Table S1.

**TABLE 1:** Demographics summary of study subjects.

	HC	SCZ	*p* Value
	(*N* = 77)	(*N* = 66)	
Age			0.973
Mean (SD)	32.1 (10.4)	32.0 (10.7)	
Median [Min, Max]	28.0 [20.0, 63.0]	29.0 [17.0, 56.0]	
Gender			0.716
Female	48 (62.3%)	44 (66.7%)	
Male	29 (37.7%)	22 (33.3%)	
Disease duration (years)			NA
Mean (SD)	NA	9.07 (7.99)	
Median [Min, Max]	NA	6.25 [0.500, 32.3]	
Missing	77 (100%)	5 (7.6%)	
CPZ equivalent			NA
Mean (SD)	NA	423 (277)	
Median [Min, Max]	NA	413 [0, 1160]	
Missing	77(100%)	7 (10.6%)	
BACS composite *Z*‐score			<0.001
Mean (SD)	−0.221 (0.932)	−1.82 (1.46)	
Median [Min, Max]	−0.163 [‐3.64, 2.27]	−1.55 [−7.67, 0.723]	
Total PANSS score			<0.001
Mean (SD)	31.8 (2.79)	49.2 (15.7)	
Median [Min, Max]	31.0 [30.0, 50.0]	44.5 [30.0, 100]	

The table shows the main information for healthy control and individuals with SCZ. The *p* values were calculated using Student's *t*‐test for continuous variables, and chi‐square test for categorical variables.

### 2.2 Basic Characteristics of Cell‐Free 5mC and 5hmC in SCZ

**FIGURE 1:**
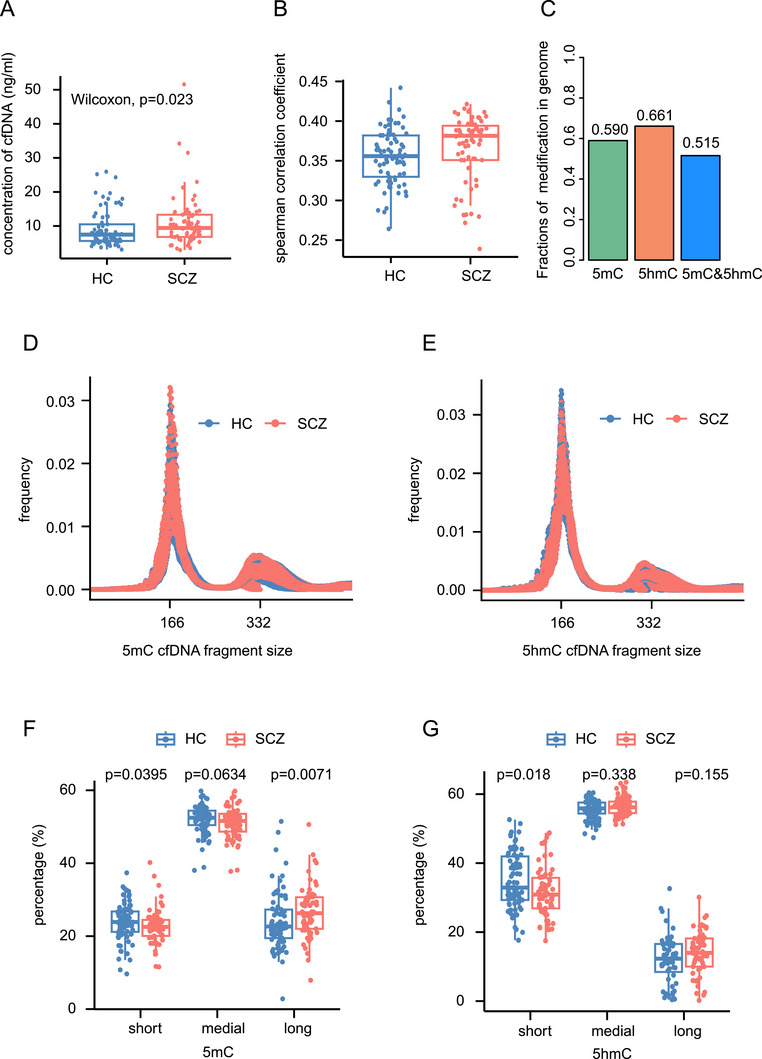
Description of the cell‐free 5‐methylcytosine (5mC) and ‐hydroxymethylcytosine (5hmC) characteristics in SCZ. (A) Comparison of plasma cell‐free DNA (cfDNA) concentrations in individuals with schizophrenia (SCZ) and healthy controls (HCs). The Wilcoxon test was used to calculate the *p* value. (B) Spearman correlation coefficient of genome‐wide scale 5mC and 5hmC modification in individuals with SCZ and HCs. (C) Fractions of 5mC, 5hmC, and 5mC and 5hmC modifications in the whole genome. (D and E) The distribution of cell‐free 5mC and 5hmC fragment sizes. The 166 bp length corresponds to DNA wrapped around a nucleosome plus linker sequence. (F and G) Comparison of the percentages of short (<166 bp), medial (166‐332 bp), and long (>332 bp) cfDNA fragments in SCZ patients and HCs. The Wilcoxon test was used to calculate the *p* value.

Consistent with previous research results [21, 22], we observed significantly greater cfDNA concentrations in the SCZ group than in the HC group (Figure 1A; Wilcoxon test, *p* value = 0.023, Power = 0.42). For each subject, we carried out cfMeDIP‐seq and hMe‐Seal to identify whole genome cfDNA 5mC and 5hmC modifications, respectively. Both 5mC and 5hmC sequencing data revealed highly specific enrichment and adequate fragments for subsequent analysis (Figure S1A–D). We then quantified the cfDNA 5mC and 5hmC modifications in a 500 bp sliding window with a 250 bp step to further compare the 5mC and 5hmC modification levels. In general, there was a moderate correlation between these two types of epigenetic modifications in both the HC and SCZ groups (Figure 1B), indicating their distinct distributions at the genome‐wide level. After filtering out windows with very low coverage, we obtained 6,026,074 windows in 5mC and 7,070,624 windows in 5hmC, covering 59.0 and 66.1% of the genome, respectively, and nearly half of the genome contained both 5mC and 5hmC modifications (Figure 1C).

In normal cells, nucleosome‐bound DNA exhibits higher methylation levels compared with flanking DNA, facilitating tighter wrapping around nucleosomes. This configuration often leads to cleavage by caspases at the linker regions between nucleosomes, resulting in predominant cfDNA fragments of approximately 167 bp [34]. Our study corroborates this finding, as we observed that the lengths of cfDNA modified by 5mC and 5hmC typically peaked at 166 bp (Figure 1D,E). In contrast, cells from SCZ patients may exhibit disrupted patterns of methylation and hydroxymethylation. Furthermore, factors such as cell cycle disturbances and altered proliferation could engage multiple cleavage sites, leading to the generation of DNA fragments of varying lengths [34]. This variability accounts for the significant differences observed in cfDNA fragment lengths between SCZ and HCs. Specifically, we found that the percentage of shorter cfDNA fragments was lower in individuals with SCZ compared with HCs (5mC: *p* value = 0.0395, power = 0.27, effect size = −0.23; 5hmC: *p* value = 0.018, power = 0.68, effect size = −0.43) (Figure 1F,G). Additionally, the percentage of longer cfDNA fragments was higher in SCZ patients than in HCs in 5mC (Figure 1F; p value = 0.007, power = 0.51, effect size = 0.35). These findings suggested that the epigenetic modifications may affect cleavage sites for caspases and therefore alter the distribution of cfDNA fragment length in SCZ.

### 2.3 Identifying SCZ‐Associated Differential 5mC and 5hmC Modification Regions

**FIGURE 2:**
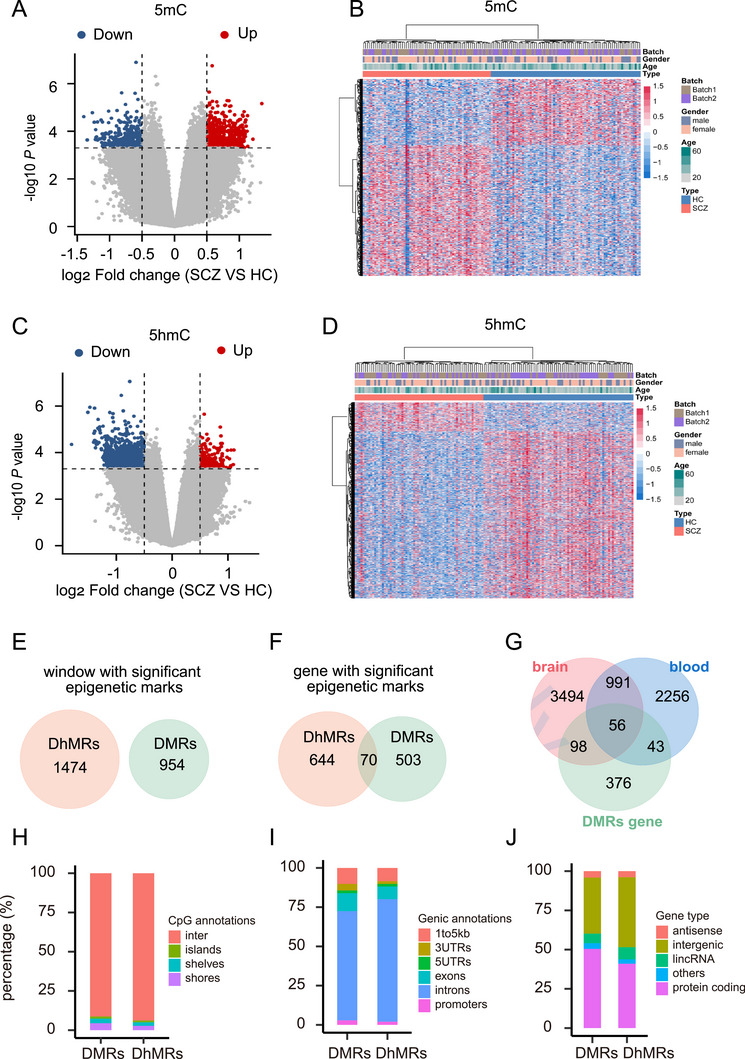
Differential 5‐methylcytosine (5mC) and 5‐hydroxymethylcytosine (5hmC) modifications between individuals with schizophrenia (SCZ) and healthy controls (HCs). (A) Volcano plot of differentially methylated regions (DMRs) between individuals with SCZ and HCs. DMRs were selected using a threshold of *p* value < 0.0005 (horizontal dotted line) and absolute log_2_ (fold change) > 0.5 (vertical dotted line). (B) Heatmap of DMRs between the SCZ and HC cohorts. (C) Volcano plot of differentially 5hmC hydroxymethylated regions (DhMRs) in individuals with SCZ and HCs. DhMRs were selected using a threshold of *p* value < 0.0005 (horizontal dotted line) and absolute log_2_ (fold change) > 0.5 (vertical dotted line). (D) Heatmap of DhMRs between the SCZ and HC cohorts. (E and F) Comparison of DMRs and DhMRs windows (E) and genes (F). (G) Comparison between the DMR genes identified in this study and genes linked to previously identified differentially methylated positions in blood and brain tissues. (H) The CpG annotations of DMRs and DhMRs. CpG shores are defined as 2 kb upstream/downstream from the ends of the CpG islands; CpG shelves are defined as another 2 kb upstream/downstream of the farthest upstream/downstream limits of the CpG shores; and the remaining genomic regions make up the inter‐CGI annotation. (I) Distribution of DMRs and DhMRs across genomic loci. A promoter was defined as the sequence less than 1 kb upstream of the transcription start site. (J) The gene types containing DMRs and DhMRs.

We compared the cell‐free 5mC and 5hmC modifications in the SCZ and HC samples in the sliding windows in section *Materials and Methods*). We performed t‐distributed stochastic neighbor embedding (t‐SNE) analysis and observed a sequencing batch effect for 5mC but not for 5hmC (Figure S2A–H). For 5mC, we detected 954 differentially methylated regions (DMRs) with age, gender, and experimental batch adjusted (321 hypo (down)‐DMRs and 633 hyper (up)‐DMRs, *p* value <0.0005 and absolute log_2_(fold change) > 0.5; Table S2). For 5hmC, we detected 1474 differentially hydroxymethylated regions (DhMRs) with age, gender adjusted (1255 hypo (down)‐DhMRs and 219 hyper (up)‐DhMRs, *p* value <0.0005 and absolute log_2_(fold change) > 0.5; Table S3). The SCZ and HC samples could be clearly separated by hierarchical clustering using the 954 DMRs and 1474 DhMRs identified, and no clear association with batch features was observed (Figure 2A–D), suggesting that we were able to identify potential epigenetic signatures to distinguish SCZ samples from HC samples. The power analyses indicating a 100% statistical power to detect DMRs and DhMRs, thereby affirming the robustness of our experimental design and statistical methods for identifying these regions. Analysis of the locations of the DMRs and DhMRs revealed no overlap between them (Figure 2E). However, at the gene level, 70 genes that contained both DMRs and DhMRs were identified (12.2% of DMR genes (*n* = 573) and 9.8% of DhMR genes (*n* = 714)) (Figure 2F). Among these, 48 genes exhibited a predominant colocalization of DMRs and DhMRs within intronic regions. This suggests a potential regulatory role for these two epigenetic modifications in intronic areas, possibly influencing splicing or transcriptional regulation. In the remaining 22 genes, DMRs and DhMRs were found in different regions of the same gene. For example, in *NCKAP1L*, DMRs were located in the promoter region, while DhMRs were situated in the intron. This indicates a possible interplay between promoter methylation and intronic hydroxymethylation in the regulation of gene expression. We then compared the DMR genes with those previously identified to include differential CpGs in brain and blood tissue [9, 10, 18, 35]. We identified 99 and 154 overlapping genes between our findings and those in peripheral blood cells (Figure 2G) and brain tissue (Figure 2G), respectively. Although these overlapping genes is limited, the hypergeometric test indicated a significant intersection (cfDNA‐brain tissue, *p* value = 9.5e−42; cfDNA‐peripheral blood, *p* value = 1.7e−22), suggesting that the 5mC DNA methylation signatures in the brain and cell‐free 5mC in blood were partially consistent. Evaluation of DMRs and DhMRs across genomic loci indicated that they were preferentially located in CpG inter‐regions and introns, and that most of them were annotated to protein‐coding genes and intergenic regions (Figure 2H–J).

**FIGURE 3:**
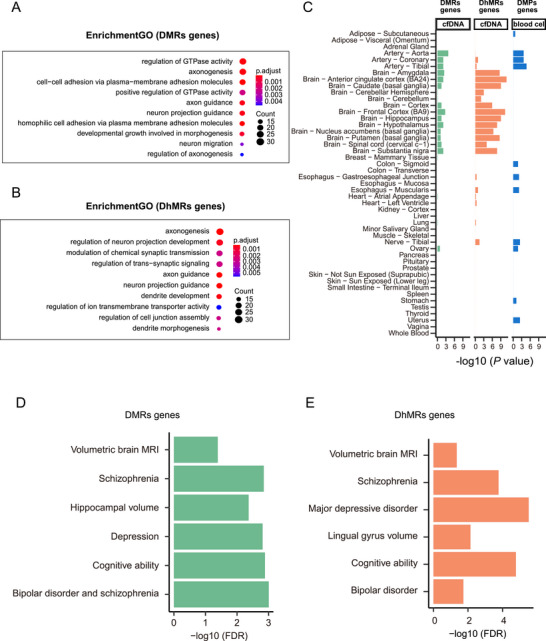
Gene set enrichment of differentially methylated regions (DMRs) and differentially hydroxymethylated regions (DhMRs) genes. (A and B) Significantly enriched Gene Ontology (GO) terms for DMR (A) and DhMR (B) genes (hypergeometric test; Benjamini‒Hochberg adjusted *p* value < 0.05). (C) Tissue‐specific enrichment analysis results of DMR and DhMR genes, as well as previously identified differentially methylated position (DMP)‐related genes in peripheral blood cells (Fisher's exact test). (D and E) Significantly enriched phenotypes of DMR (D) and DhMR (E) genes according to the datasets deposited in the FUMA_GWAS_ (hypergeometric test; Benjamini–Hochberg adjusted *p* value < 0.05).

Furthermore, GO enrichment analysis revealed that genes with DMRs and DhMRs were both significantly enriched for several terms involved in neuronal function (Figure 3A,B; BH‐adjusted *p* value < 0.05), including axonogenesis, axon guidance and neuron projection guidance, supporting the hypothesis that SCZ has a neurodevelopment origin [36]. In addition, DhMR genes were associated with synaptic biological processes, such as the modulation of chemical synaptic transmission and the regulation of trans‐synaptic signaling, which were highlighted by common and rare genetic variants enrichment results [5, 37]. Moreover, we performed tissue‐specific enrichment analysis based on bulk RNA‐seq from GTEx to identify the tissues most relevant for DMR and DhMR genes. Compared with a previous study in which differentially methylated position (DMP) genes were identified in peripheral blood cells [18], The DMR gene enrichment differed between brain and nonbrain tissues, and a similar but clearer contrast was observed for the DhMR genes (Figure 3C). As mentioned above, cfDNA inherently contains DNA derived from peripheral blood but also encompasses DNA from other solid tissue sources, for example, brain tissues. Therefore, our DMR and DhMR signatures, which can differentiate SCZ from HC, may include brain‐derived DNA from brain tissues. Consequently, DMR‐ and DhMRs‐genes present obviously enrichment in brain tissues. In contrast, peripheral blood cells consist of granulocytes, monocytes, T lymphocytes, and B lymphocytes, meaning that genomic DNA isolated from peripheral blood cells is unable to capture the brain‐specific signatures.

**FIGURE 4:**
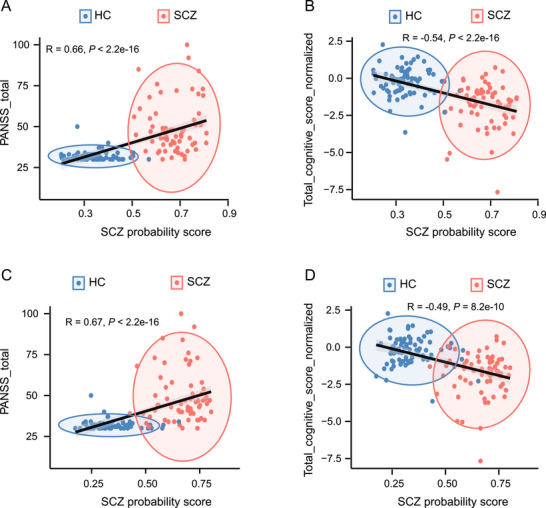
Correlation between the clinical severity score and schizophrenia (SCZ) probability score obtained from the gradient boosting machine (GBM) model. (A and B) Spearman correlation between SCZ probability scores from the differentially methylated regions (DMRs)‐GBM model and clinical severity scores, including positive and negative syndrome scale (PANSS) total scores (A) and cognitive total scores, normalized by age and gender (B). (C and D) Spearman correlation between the SCZ probability score from the differentially hydroxymethylated regions (DhMRs)‐GBM model and the clinical severity scores, including the PANSS total score (C), and the total cognitive score normalized by age and gender (D).

We next sought to interrogate the relationship between epigenetic modifications and clinical symptoms. We developed GBM models based on DMRs and DhMRs to predict clinical severity. Each participant was scored based on the response probability from the models. Higher values indicate a greater probability of SCZ. The probability scores were significantly positively correlated with the PANSS total score but negatively correlated with the normalized cognitive score (Figure 4A–D), indicating that DMRs and DhMRs are clinically relevant signatures.

### 2.4 GWAS Enrichment and Colocalization Analysis

To further investigate the functionality of the DMR and DhMR genes, we carried out a GWAS enrichment analysis using the FUMA dataset [38]. Both the DMR and DhMR genes were enriched in psychiatric domains, including depression, cognitive, bipolar disorder, and SCZ (Figure 3D,E; BH‐adjusted *p* value < 0.05). Additionally, DMR and DhMR genes were also enriched in brain volume phenotypes. For example, DMR genes were enriched for hippocampal volume and volumetric brain MRI, and DhMR genes were enriched for lingual gyrus volume and volumetric brain MRI. These results indicated that the DMR and DhMR genes are associated with both SCZ and brain MRI phenotypes.

Based on the above results, we further investigated the relation between SCZ GWAS and DMRs as well as DhMRs. GWAS have identified SCZ‐associated 342 SNPs at 287 distinct genomic loci [5], but further functional interpretation is needed. Our analysis revealed that 43 DMRs colocalized with 50 SCZ risk SNPs (Table S4), and 75 DhMRs colocalized with 71 SCZ risk SNPs (Table S5). Simulation analyses indicated that the distance between DhMRs and colocalized SNPs was significantly shorter than expected by chance (Wilcoxon test, *p* value < 2.2e−16). In contrast, this trend was not observed for DMRs, suggesting that 5mC modifications may not have a direct association with SCZ SNPs. Instead, 5hmC appears to play a more direct role in the genetic regulation associated with SCZ. In addition, cfMeDIP‐seq and hMe‐Seal methods do not require DNA transformation and therefore have potential advantages for variant analysis. We identified 21 and 26 SCZ‐specific variants in 5mC and 5hmC, respectively, that had positions, mutation types, and directions identical to those of the SCZ SNPs with the greatest significance at the genomic loci (Tables S6 and S7). Similarly, several of these SNPs are near DMRs and DhMRs. For example, SNP rs11227250, which has a C to T mutation, is near the DMR region (chr11:65418250‐65418750) (Figure S3A); SNP rs61405217, which has a C to T mutation, is near the DhMR region (chr4:170473000‐170473500) (Figure S3B). These results provided clues that genetic background may influence some SCZ‐related DMRs and DhMRs.

### 2.5 Association of cfDNA Epigenetic Changes with Brain Structural Abnormalities

Compared with HCs, individuals with SCZ showed widespread atrophy in cortical and subcortical regions (Figure S4A; BH adjusted *p* value < 0.05). A total of 22 regional measures were decreased (Tables S8 and S9), including: cortical thickness (CT) of the bilateral frontal and temporal lobes; cortical volume (CV) of the bilateral superior frontal gyrus (SFG) and right medial orbitofrontal gyrus (mOFG); surface area (SA) of the left SFG; and subcortical volume (SV) of the bilateral cornu ammonis (CA) 1, molecular layer (ML) and the right GC_ML_DG of the hippocampus, the right medial pulvinar (PuM) nucleus of the thalamus, and the right amygdala. Although brain atrophy in individuals with SCZ was well defined in our study, hierarchical clustering showed that these altered region measures could not distinguish HCs and individuals with SCZ (Figure S4B).

Brain atrophy may reflect brain cell apoptosis and disruption of the blood‒brain barrier, which could contribute to changes in cfDNA epigenetic modifications in individuals with SCZ. We therefore investigated the associations of the 954 DMRs and 1474 DhMRs with all brain image structures (*n* = 305) by univariate (Spearman rank correlation analysis) and multivariate statistical analysis (sparse canonical correlation analysis (sCCA)), respectively (see section *Materials and Methods*). The brain regions that were identified by both methods were considered reliably correlated with cfDNA.

**FIGURE 5:**
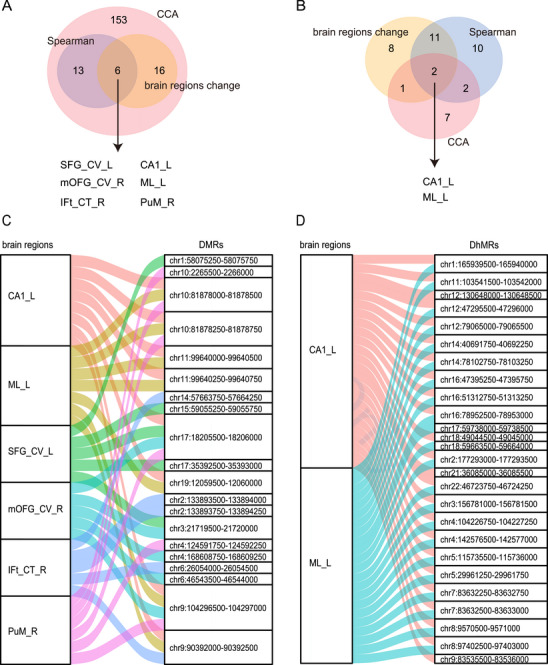
Associations between epigenetic changes and brain structure. (A and B) Venn diagram representing the overlapping brain regions that showed changes in individuals with schizophrenia (SCZ) compared with healthy controls (HCs) and were associated with differentially methylated regions (DMRs) (A) or differentially 5hmC hydroxymethylated regions (DhMRs) (B) according to Spearman rank correlation and sparse canonical correlation (CCA) analysis methods. (C and D) The alluvial plot shows the significant and reliable correlations between brain regional measures and DMRs (C) as well as DhMRs (D). cortical volume of the left superior frontal gyrus: SFG_CV_L; cortical volume of the right medial orbitofrontal gyrus: mOFG_CV_R; left cornu ammon 1: CA1_L; left molecular layer: ML_L; right medial pulvinar nucleus of the thalamus: PuM_R; cortical thickness of the pars triangularis region of right inferior frontal gyrus: IFt_CT_R.

For 5mC (Figure 5A), we identified 19 brain regions that were significantly correlated with DMRs based on Spearman rank correlation analysis. In contrast, sCCA was utilized to directly examine the relationships between the two data matrices: DMRs and brain image structures. As a result, a total of 622 DMRs and 188 brain regional measures accounted for the sCCA correlation. We then overlapped the results from Spearman rank correlation (*n* = 19) and sCCA (*n* = 188) as well as different brain region structural measures (*n* = 22) between SCZ and HCs. We found that 19 brain regions were significantly and reliably correlated with DMRs, of which six regions also showed significant differences in atrophy between HCs and individuals with SCZ. Similarly, for 5hmC (Figure 5B), we identified 25 brain regions that were significantly correlated with DhMRs based on Spearman rank correlation analysis. Additionally, a total of 72 DhMRs and 12 brain regional measures accounted for the sCCA correlation. We then overlapped the results from Spearman rank correlation (*n* = 25) and sCCA (*n* = 12) as well as different brain region structural measures (*n* = 22) between SCZ and HC. As a result, four brain regions were found to be significantly and reliably correlated with DhMRs, of which two regions also showed significant differences in atrophy between HCs and individuals with SCZ. In summary, the atrophy of some regions in the hippocampus, thalamus, and frontal lobe was related to cfDNA epigenetic modification in individuals with SCZ, of which the left CA1 region and ML of the hippocampus were associated with both DMRs and DhMRs.

We further assessed the DMR/DhMR–brain region association pairs arising from the Spearman rank correlation analysis. For 5mC, there were 36 associations between six brain structure regions and 20 DMRs (Figure 5C). Notably, the methylation of DMR (chr1: 58075250–58075750; located on *DAB1*) was correlated with the volume of the left SFG. *DAB1* is a protein‐coding gene that is widely reported to be associated with SCZ [39]. Dysfunction of Reelin–Dab1 signaling can lead to behavioral deficits in individuals with SCZ and bipolar disorder [40]. For 5hmC, there were 46 associations between two brain structure regions and 25 DhMRs (Figure 5D). Typically, hydroxymethylation of DhMR (chr7:83632250‐83632750; located on *SEMA3A*) is correlated with the left CA1 region. *SEMA3A* is involved in axon guidance and regulates dendritic attraction [41]. Expression of *SEMA3A* is increased in the brain of individuals with SCZ, which might lead to the synaptic pathology observed in individuals with the disease [42].

## 3 Discussion

This is the first parallel investigation of genome‐wide 5mC and 5hmC levels in cfDNA from individuals with SCZ. First, we identified 954 DMRs and 1474 DhMRs with distinct epigenetic modifications in individuals with SCZ compared with HCs; many DMRs and DhMRs were located in genes specifically expressed in brain tissues and enriched in neuronal functions. Second, GWAS enrichment analysis indicated that these DMR and DhMR genes were associated with both psychiatric and brain volume phenotypes. Additionally, colocalization analysis revealed that DMR and DhMR locations overlapped with GWAS‐identified genomic loci of SCZ. Finally, we observed associations between brain structure atrophy and altered DMRs and DhMRs, especially in the hippocampus. Together, these results indicated that cell‐free 5mC and 5hmC studies contribute to identifying potential SCZ signatures and understanding its pathogenesis.

Previous DNA methylation studies of SCZ were performed in postmortem brain tissue [9, 10], which is difficult to acquire and does not reflect the state of the brain in vivo. In contrast, peripheral blood is more easily accessible. Although some studies have indicated that methylation patterns in peripheral blood cells are related to the methylation patterns observed in brain tissues [17], brain cell apoptosis and blood‒brain barrier disruption may lead to the release of brain‐derived cfDNA into plasma. This cfDNA may provide more information about the biological processes in SCZ brain tissues than DNA in peripheral blood cells. For example, the DMR and DhMR genes identified in this study both showed differential enrichment in brain and nonbrain tissues, while this result was not observed for the DMP genes identified from blood cells (Figure 3C). In addition, GO term enrichment analysis revealed that neuronal functions were linked to the DMR and DhMR genes. Moreover, the DMR genes included one neuron‐specific gene (*PHF21B*) and one oligodendrocyte‐specific 5mC gene (*KIF13A*), which were identified from 39 cell types via deep whole‐genome bisulfite sequencing [43], and the DhMR genes included two brain‐specific 5hmC genes (*CDH4* and *TMEM132B*), which were identified across 19 tissue types via hmC‐CATCH [44]. These results provide basic evidence that cfDNA possesses brain‐derived signatures that could be used as a biomarker to distinguish individuals with SCZ from HCs (Figure 2B,D).

Moreover, our brain structure correlation analysis further validated that these DMRs and DhMRs were associated with brain volume phenotypes, especially in the hippocampus. Many studies have demonstrated that SCZ‐related gene dysfunction is localized to hippocampal glutamatergic neurons [5, 37]. SCZ‐related DNA methylation has also been detected in the hippocampus [45]. Consistent with these findings, our results showed that the hippocampal CA1 and the ML are the regions most susceptible to the changes of epigenetic modification. We also found that atrophy in multiple regions (mOFC, SFG, and IFt) of the frontal lobe was associated with DMRs. Based on the findings from magnetic resonance spectroscopy [46], structural MRI [47], and molecular imaging [48], functional and structural abnormalities of the frontal lobe are considered to be robust phenomena in individuals with SCZ. Specifically, a thicker mOFC is related to greater schizotypy in the general population [49]; the degree of volume reduction of the SFG is determined by the genetic influence of SCZ [50]; and a reduced IFt thickness is associated with increased polygenic risk scores for SCZ [51]. Exploring the relationship between epigenetic modifications and brain structure might extend our understanding of the role of genes in SCZ development.

There are several limitations in our study. The sample size of our cohort was relatively small, and the cell‐free signatures need to be validated in external independent cohorts. In addition, changes in 5mC and 5hmC may be influenced by the long‐term effects of drugs and disease course, and further research should focus on first‐episode drug‐naïve individuals with SCZ. Moreover, individuals with other psychotic diseases with overlapping clinical and neurobiological features, including bipolar disorder and major depressive disorder, should also be included to identify SCZ‐specific signatures. Although we detected brain‐specific genes associated with cell‐free 5mC and 5hmC, the current deconvolution algorithms were designed for DNA methylation arrays or whole‐genome bisulfite sequencing; therefore, we did not use a deconvolution algorithm to estimate the contribution of different tissues, particularly brain tissue, to the levels of cell‐free 5mC and 5hmC in plasma.

In summary, in this study, we identified a panel of DMRs and DhMRs that showed potential for SCZ detection. The analysis of DMRs and DhMRs as well as brain structure provided multiple insights into SCZ pathogenesis. In future studies we will recruit larger cohorts to validate our results.

## 4 Methods and Materials

### 4.1 Participants and Sample Collection

Individuals with SCZ were enrolled from the mental health clinic of West China Hospital of Sichuan University between December 2021 and October 2022. SCZ diagnoses were confirmed by an experienced psychiatrist using DSM‐5 criteria. All of the patients were taking antipsychotic medicine regularly as advised by their doctors. The disease duration was assessed through the Nottingham Onset Schedule [52] with information provided by individuals with SCZ, their family members and clinical documentation, and the disease duration of each patient was calculated. Antipsychotic dosages were standardized using chlorpromazine equivalents (CPZ equivalent) [53]. HCs were recruited through advertisements, and all of them and their siblings had no history of psychosis. The general exclusion criteria for individuals of SCZ and HCs included brain injury history, serious disease conditions, drug or alcohol abuse, and brain abnormalities observable in T2‐weighted images. Finally, we included 66 individuals with SCZ and 77 HCs in this study.

The severity of psychiatric symptoms in individuals with SCZ was assessed using the PANSS [54]. Specifically, through structured interviews, the total PANSS score was the sum of the positive, negative, and general psychopathology scores. A higher PANSS score indicates more serious psychiatric symptoms. Moreover, we also assessed the cognitive level of each participant using another scale called the BACS [55], which assessed the cognitive levels from six dimensions, including verbal learning, working memory, motor speed, verbal fluency, attention and speed of information processing, and executive function. Then, we calculated standard scores for each dimension and the composite *Z*‐score. A lower composite *Z*‐score indicates a lower level of cognition.

### 4.2 Blood Sample Processing, cfDNA Extraction, and Sequencing

Blood was collected in Ethylenediaminetetraacetic Acid (EDTA) tubes. Plasma was separated within 2 h by centrifugation. cfDNA was extracted with a VAHTS Serum/Plasma Circulating DNA Kit and quantified by a Qubit fluorometer. For library construction, 10–30 ng of cfDNA was used, with adaptor ligation including spike‐in controls. The spike‐in control included three lambda DNA products: one unmodified and two modified with 5mC and 5hmC. Subsequently, cfDNA sequencing for 5mC and 5hmC was performed using the cfMeDIP‐seq and selective chemical labeling (hMe‐Seal) methods, as detailed in the previous study [56].

### 4.3 CfDNA Sequencing Data Processing

The cfDNA 5mC‐ and 5hmC‐captured libraries were sequenced using the Illumina NovaSeq 6000 platform (150 bp paired‐end runs). Low‐quality reads were filtered with Fastp [57] software. Reads were then aligned to hg19 and spike‐in DNA using BWA [58] (version 0.7.17‐r1188). SAMtools (version 1.7) [59] filtered SAM files (parameters: ‐f 3 ‐F 3852 ‐q 30) to retain high‐quality and properly paired reads, which were converted to BAM format. Picard (version 2.25.5) sorted and removed duplicate reads from BAM files. Bedtools (version 2.26.0) converted BAM to BED format. The paired reads aligned to the same autosome in the correct orientation with an insert size of 20–1000 bp were used for further analysis. Capture efficiency for cfDNA 5mC and 5hmC was calculated as the ratio of reads aligned to type‐specific spike‐in DNA to total spike‐in DNA reads.

### 4.4 Identification of DMRs and DhMRs

We utilized a 500 bp sliding window with a 250 bp step to scan each autosomal chromosome and then calculated the 5mC or 5hmC read coverage in each window using bedtools with the “‐coverage” parameter. Windows overlapping with low average mappability scores (<0.9) and dark regions that included centromeres, telomeres, and DAC and Duke excluded regions from the UCSC Table Browser, were removed. The mappability score was calculated using the GEM mappability program with 150‐mer length on the hg19 reference genome. In addition, only windows with more than three counts in at least 10 samples across all HC and SCZ samples (nonlow coverage windows) were retained for subsequent differential analysis. To assess potential batch effects in our dataset, we employed t‐distributed stochastic neighbor embedding (t‐SNE), a dimensionality reduction technique particularly effective for visualizing high‐dimensional data. The analysis was conducted following these steps: The raw counts of genomic windows were initially transformed using the variance‐stabilizing transformation (VST) function provided by R package DESeq2, the transformation produces transformed data on the log2 scale, which has been normalized with respect to library size or other normalization factors, making the data more suitable for t‐SNE analysis. Then, we performed t‐SNE with transformed data as input using the *Rtsne* function provided by R package Rtsne, which constructed a two‐dimensional embedding of transformed high‐dimensional data. The results of the t‐SNE analysis were then visualized using a scatter plot, where each point represents a sample. The resulting t‐SNE plot allowed us to visually assess the presence of batch effects. The raw window counts were used as input for DESeq2 to detect DMRs or DhMRs. For 5mC, age, gender, and experimental batch factors were included in the design model; for 5hmC, age and gender were included in the design model. Windows with *p* values < 0.005 and absolute fold changes greater than 0.5 were considered DMRs (*n* = 954) and DhMRs (*n* = 1474).

We applied gradient boosting machine (GBM) algorithms to the normalized counts by the DESeq2 *vst* function to develop a prediction model for DMRs and DhMRs, respectively. For every participant, GBM returned a disease score ranging from 0 to 1. A higher score indicates a greater probability of SCZ disease. The hyperparameters of the GBM model were selected using the R caret package with 10 rounds of threefold cross‐validation.

### 4.5 Annotation of DMRs and DhMRs and Enrichment Analysis

The CpG and genic annotations of DMRs and DhMRs were performed using the R package annotatr [60]. The DMRs or DhMRs were annotated to gene region locations and gene types that were extracted from the gtf file (https://ftp.ebi.ac.uk/pub/databases/gencode/Gencode_human/release_19/gencode.v19.annotation.gtf.gz). The R package clusterProfiler was used to identify significantly enriched GO terms for DMR genes and DhMR genes (two‐sided hypergeometric tests, BH adjusted *p* value < 0.05). The background group of genes consisted of all genes (*n* = 18,862) listed in the human GO database. The R package *deTS* was used to conduct tissue‐specific enrichment analysis. The background gene group included a panel of 14,725 protein‐coding, nonhousekeeping genes in 47 tissues obtained from GTEx RNA‐seq data. GWAS enrichment analysis was performed to assess the likelihood of gene overlap between the DMRs/DhMRs and published GWAS results. The analysis was performed using the FUMA website (https://fuma.ctglab.nl/) with the GENE2Function tool. The FUMA dataset can be download in this website: https://fuma.ctglab.nl/downloadPage. The gene set we used to performed GWAS enrichment analysis was based on MSigDB and GWAS catalog gene‐set files (FUMA version 1.5.2).

### 4.6 Colocalization Analysis of DMRs and DhMRs With SCZ Genomic Loci

We conducted a colocalization analysis to identify overlapping signals between DMRs and DhMRs with SNPs (*n* = 342) previously reported to be associated with SCZ at 287 unique genomic loci [5]. We considered DMRs/DhMRs overlapping within the genomic loci of SNPs to be colocalized. To evaluate whether the proximity of colocalized neighboring SNPs could have occurred by random chance, we utilized a software tool called simuG [61] to simulate random SNPs in hg19 genome. We calculated the distances between DMRs/DhMRs and these randomized SNPs, repeating this process 1000 times to generate a distribution of expected distances. Subsequently, we compared the observed distances from our actual data to the distribution derived from the randomization by Wilcoxon test. This comparison enabled us to assess whether the observed proximity was significantly closer than what would be expected by chance.

Single nucleotide variants (SNVs) were identified in 287 unique genomic loci separately from all HCs and all SCZ bam files using BCFtools. High‐quality (>30) SNVs with a sequencing depth greater than 30 were retained. Then, SNVs whose position, mutation type, and direction were identical to those of the SCZ SNP with the highest significance in the genomic loci were retained. These SNVs called in SCZ samples but not in HC samples were considered SCZ‐specific variants.

### 4.7 Acquisition and Analysis of Brain Structural Images

Neuroimaging acquisition was conducted on a GE Healthcare Signa Premier 3.0T system utilizing a 48‐channel phased‐array head coil, with T1‐weighted images obtained through magnetization‐prepared rapid gradient echo (MPRAGE) sequence. The detailed parameters were repetition time = 9.6 ms, echo time = 2.32 ms, flip angle = 8°, field of view = 240 × 240 mm^2^, and slice thickness = 1 mm. A total of 336 axial slices with a resolution of 0.47 × 0.47 mm^2^ were obtained.

We first transformed the images in DICOM format to the Nifti format and reoriented the origin to the anterior commissure using SPM12 (https://www.fil.ion.ucl.ac.uk/spm/software/spm12/). All reoriented Nifti images were then processed by FreeSurfer (https://surfer.nmr.mgh.harvard.edu) software version 7.1 with a “recon‐all” workflow [62], including skull stripping, calculating and reconstructing the boundaries of white matter and gray matter of bilateral hemispheres, inflating the surface to the Desikan–Killiany atlas, and parcellating to independent cortical regions. The CT, CV, and SA of 68 distinct cortical regions defined by the Desikan–Killiany atlas were extracted. We further extracted SV of the caudate, putamen, pallidum, nucleus accumbens, and subregions of the thalamus, hippocampus, and amygdala, by FreeSurfer software. All measures of cortical and subcortical regions (Table S10) and intracranial volume (ICV) were obtained and used in subsequent analyses.

We implemented linear models to remove variance related to age, gender, and ICV, and the median raw score was added to the respective residual value after the correction. Since CT is not related to the ICV, only age and gender were accounted for correction. The Wilcoxon test was used to identify differences in brain structural measures in individuals with SCZ compared with HCs. Significance was defined as an FDR‐adjusted *p* value < 0.05.

### 4.8 Correlation Analysis between DMRs and DhMRs and Brain Imaging Data

We performed Spearman rank correlation analysis and sCCA to investigate the associations between the 954 DMRs and 1474 DhMRs with all brain image structures (*n* = 305). After matching the cfDNA and brain imaging data, 59 SCZ and 68 HC participants remained for further analysis. The DMR/DhMR data were regressed out of the age and gender covariates, and median DMR/DhMR values were added back to the respective residual values after correction. In the Spearman rank correlation analysis, we calculated correlation coefficients and *p* values for each DMR and DhMR in relation to brain imaging structures, resulting in 290,970 (954 × 305) correlation pairs for DMRs and brain regions, and 449,570 (1474 × 305) correlation pairs for DhMRs and brain regions. Those correlative pairs with FDR‐adjusted *p* values < 0.05 were considered significant. leading to the identification of 392 significant DMR‐brain region pairs and 935 significant DhMR‐brain region pairs. To avoid randomness, only brain regions significantly associated with ≥5 DMRs or ≥10 DhMRs were used for subsequent analysis, leaving 19 brain regions significantly associated with 43 DMRs and 25 brain regions significantly associated with 80 DhMRs.

sCCA is a classical method for examining the relationships between two types of data matrices and can be used when the number of variables exceeds the sample size. The R package PMA was used to perform sCCA on our data. The function CCA.permute was used to automatically select tuning parameters for sparse CCA, and the CCA function was used to perform sCCA analysis with three canonical vectors to be obtained. The variable with a nonzero canonical coefficient, indicating that it contributes to the overall correlation between two types of data matrices. Consequently, a total of 622 DMRs and 188 brain regional measures were identified as contributing to the sCCA correlation, whereas 72 DhMRs and 12 brain regional measures accounted for the sCCA correlation.

Finally, the brain regions that were identified by both methods were considered to reliably correlate with DMRs or DhMRs.

### 4.9 Statistical Power Analyses

In identifying SCZ‐associated differential 5mC and 5hmC modification regions, the power analysis for DMRs and DhMRs was performed. Effect size was derived from DESeq2 results by dividing the absolute *log2FoldChange* by *lfcSE*. Subsequently, we applied the *pwr.t.test* function in R package *pwr* with the calculated effect size, sample size, and a significance level of 0.005 to compute the statistical power for each region. The similar analysis was conducted for cfDNA concentrations and lengths in the context of differential analysis between HC and SCZ group.

## Author Contributions

S.L. and D.X. conceived the project. X.W., L.L., Q.Z., B.Z., J.Z., S.M.L., W.J.Z., C.Y.L., and Q.Y.G. collected the data. G.X., X.W., and W.H. performed the data analysis. G.X., X.W., and Q.N.Z wrote and edited the manuscript. All authors have read and approved the final manuscript.

## Conflicts of Interest

Author Jun Zhang and Wen Hu are employees in Tailai Inc., Chengdu, but have no potential relevant financial or nonfinancial interests to disclose. The other authors have no conflicts of interest to declare.

## Ethics Statement

This study adhered to national/institutional ethical guidelines and the Helsinki Declaration (1975, 2008 revision). Human subject protocols were approved by the Biomedical Research Ethics Committee of West China Hospital, Sichuan University (Approval #1016). Informed consent was obtained from all participants in the study.

## Supporting information



Supporting Information

Supporting Information

Supporting Information

Supporting Information

Supporting Information

Supporting Information

Supporting Information

Supporting Information

Supporting Information

Supporting Information

Supporting Information

## Data Availability

All 5mC and 5hmC sequencing data generated in this study are available at the Genome Sequence Archive for Humans (https://ngdc.cncb.ac.cn/gsa‐human/) with accession number: HRA007010.
