# Supplementary material for: Genome‐Wide 5‐Methylcytosine and 5‐Hydroxymethylcytosine Signatures Analysis of Plasma Cell‐Free DNA in Schizophrenia
Source: MedComm (2020). 2025 Jul 30;6(8):e70293. doi: 10.1002/mco2.70293 (PMC12308070; doi:10.1002/mco2.70293)
Supplement: Supplementary file 11 — Supporting Information [file MCO2-6-e70293-s006.docx]

**Title:** **Genome-wide 5-methylcytosine and 5-hydroxymethylcytosine signatures analysis of plasma cell-free DNA in schizophrenia**

Running title: Cell-free epigenomic signatures of SCZ

Gang Xue^1#^, Xia Wei ^2,3#^, Li Li^4^, Qi Zhang ^2,3^, Shanming Liu^5^, Jun Zhang^6^, Wen Hu^6^, Qiannan Zhao^2,3^, Wenjing Zhang^2,3^, Chunyan Luo^2,3^, Qiyong Gong^2,3^, Bo Zhang^5*^, Dan Xie^1*^, Su Lui^2,3*^

^1^Laboratory of Omics Technology and Bioinformatics, Frontiers Science Center for Disease-related Molecular Network, State Key Laboratory of Biotherapy, West China Hospital, Sichuan University, Chengdu, Sichuan, 610041, China.

^2^Department of Radiology, and Functional and Molecular Imaging Key Laboratory of Sichuan Province, West China Hospital of Sichuan University, Chengdu, China.

^3^Research Unit of Psychoradiology, Chinese Academy of Medical Sciences, Chengdu, China.

^4^Department of Nuclear Medicine, West China Hospital, Sichuan University, Chengdu, China.

^5^Mental Health Center, West China Hospital, Sichuan University, Chengdu, China

^6^Tailai Inc., Chengdu, Sichuan 200233, P. R. China.

^#^These authors contributed equally to the work.

**^*^**These authors contributed equally as co-corresponding authors.

^*^Correspondence:

Huaxi MR Research Center, West China Hospital of Sichuan University, 37 Guoxue Xiang, 610041 Chengdu, China; tel/fax: +86-28-85423960, e-mail: lusuwcums@hotmail.com (Su Lui).

Laboratory of Omics Technology and Bioinformatics, Frontiers Science Center for Disease-related Molecular Network, State Key Laboratory of Biotherapy, West China Hospital, Sichuan University, Chengdu, Sichuan, 610041, China; e-mail: danxie@scu.edu.cn (Dan Xie).

Mental Health Center, West China Hospital, Sichuan University, 610041 Chengdu, China; email: zb_73@126.com (Bo Zhang).


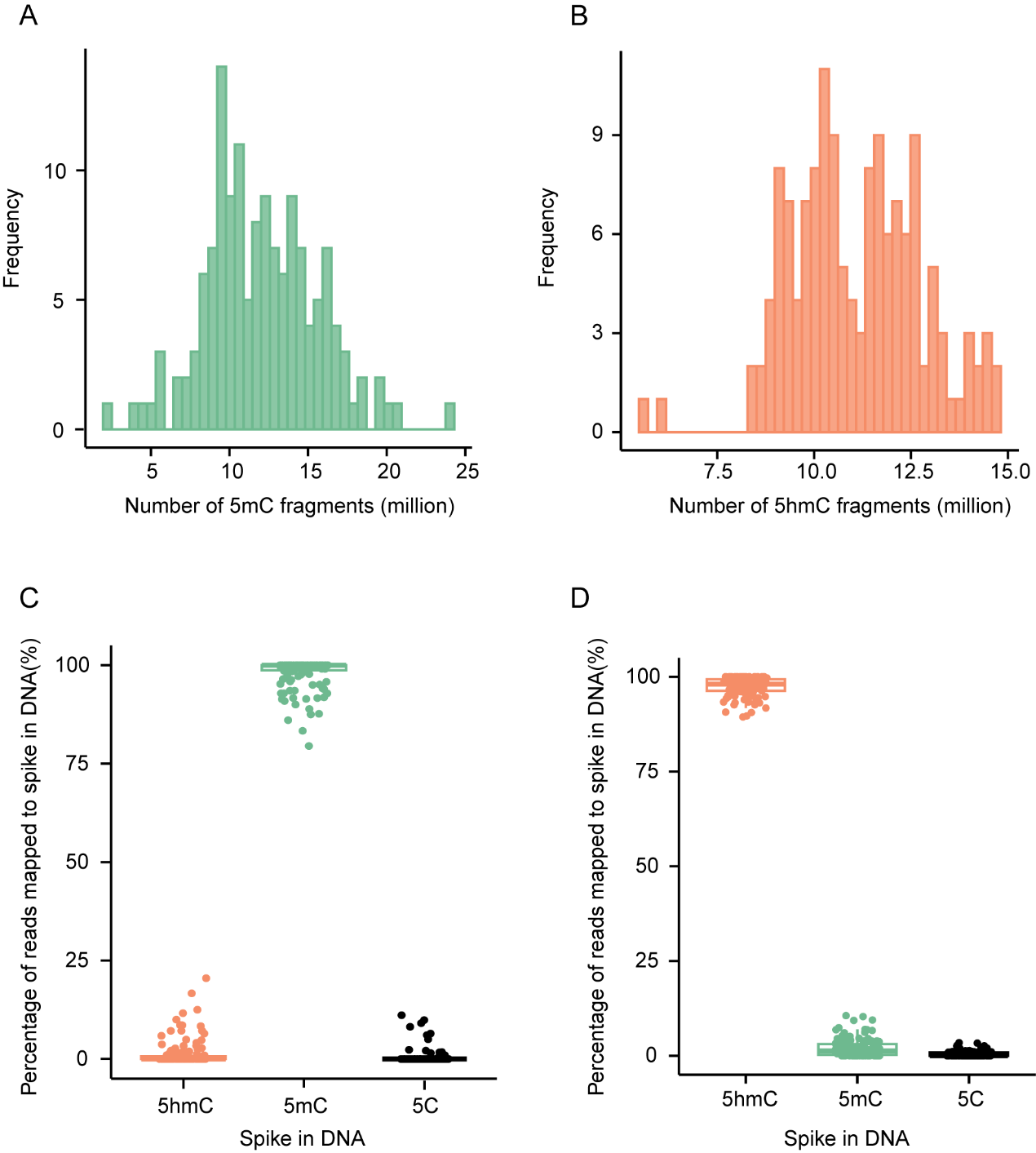


**Figure S1. Cell-free 5-methylcytosine (5mC) and 5-hydroxymethylcytosine (5hmC) data statistics. A, B,** Histogram plot of the number of high-quality fragments (paired reads) in 5mC (**A**) and 5hmC (**B**) samples (n=143). **C, D**, Percentage of 5mC (**C**) and 5hmC (**D**) reads mapped to the spike-in DNA in the sequencing libraries. The 5mC spike-in DNA is specifically enriched in the 5mC libraries (**C**). The 5hmC spike-in DNA is specifically enriched in the 5hmC libraries (**D**). Each dot in the boxplot represents a cell-free DNA sample.


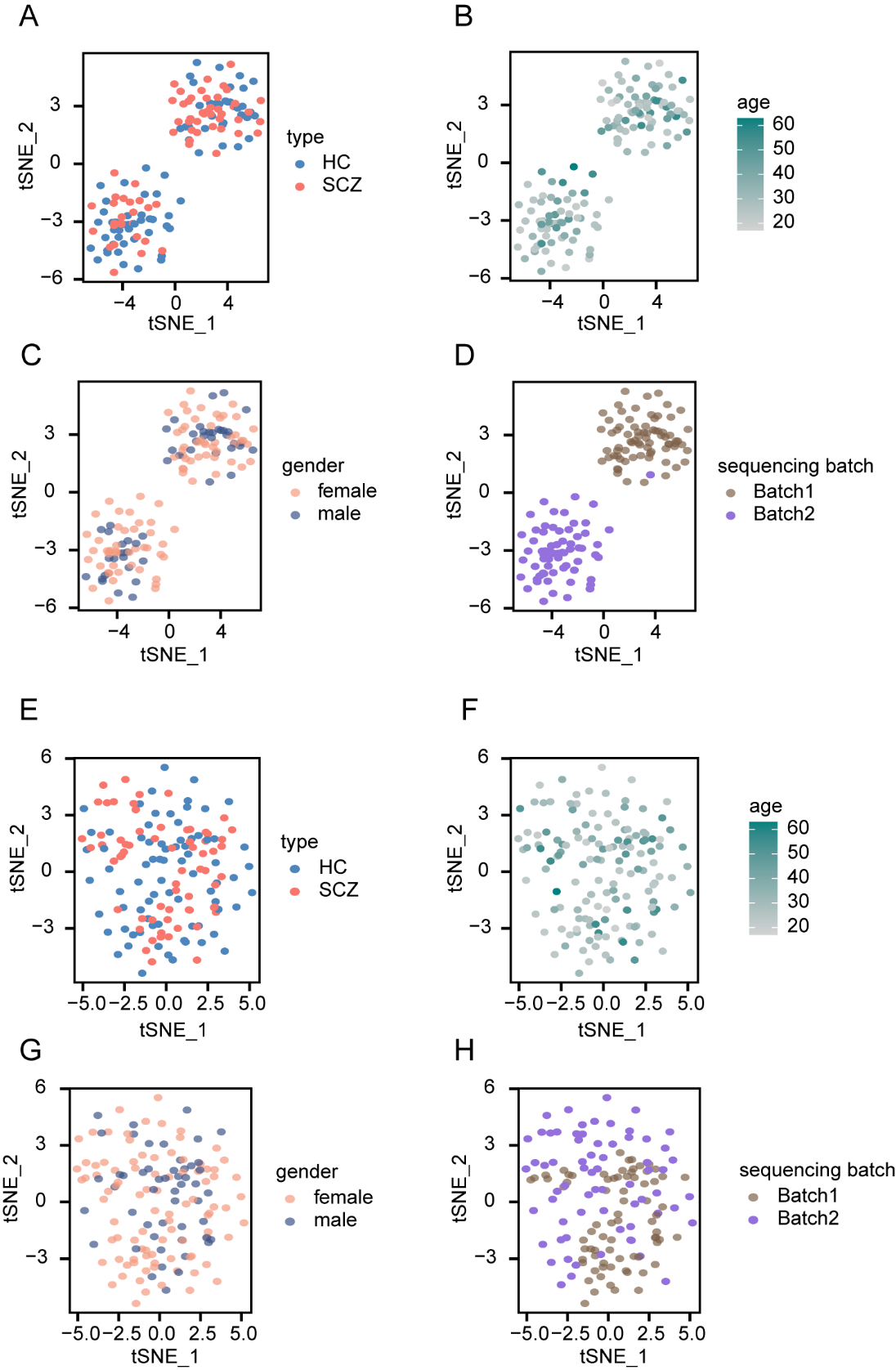


**Figure S2.** **Batch factor analysis and dimension reduction. A-D,** T-SNE plot of 5-methylcytosine (5mC) samples using normalized counts on windows (n=6,026,074) colored by type **(A)**, age **(B)**, gender **(C)** and sequencing batch **(D)**. **E-H,** T-SNE plot of 5-hydroxymethylcytosine (5hmC) samples using normalized counts on windows (n=7,070,624) colored by type **(E)**, age **(F)**, gender **(G)** and sequencing batch **(H)**.


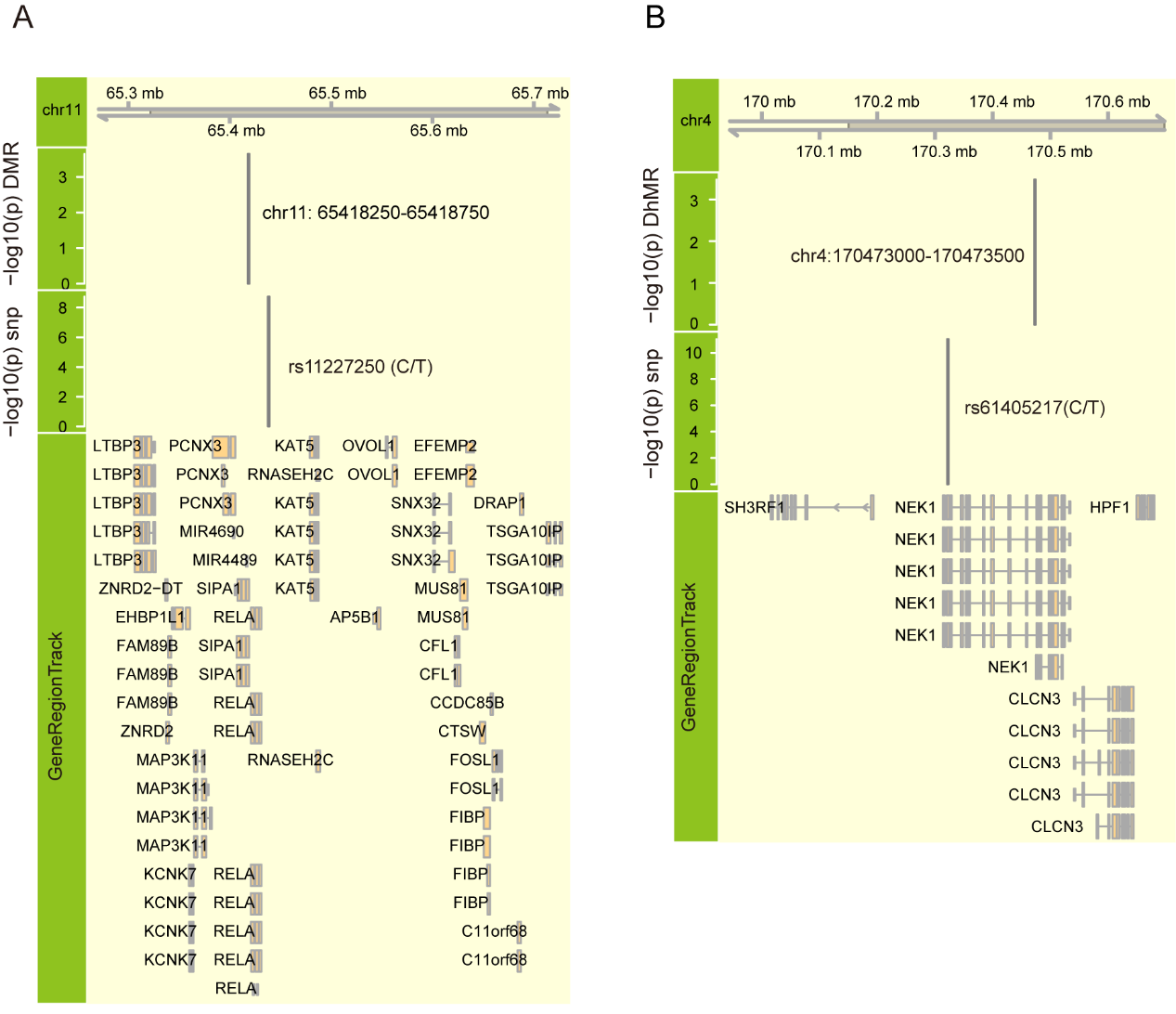


**Figure S3. Colocalization of differentially methylated regions (DMRs) and differentially hydroxymethylated regions (DhMRs) with single-nucleotide polymorphisms (SNPs)**. The vertical bars correspond to the genomic locations of DMRs (**A**) and DhMRs (**B**), respectively. Similarly, below vertical bars indicate schizophrenia (SCZ)-associated SNPs identified by GWAS. Gene locations are indicated by horizontal bars.


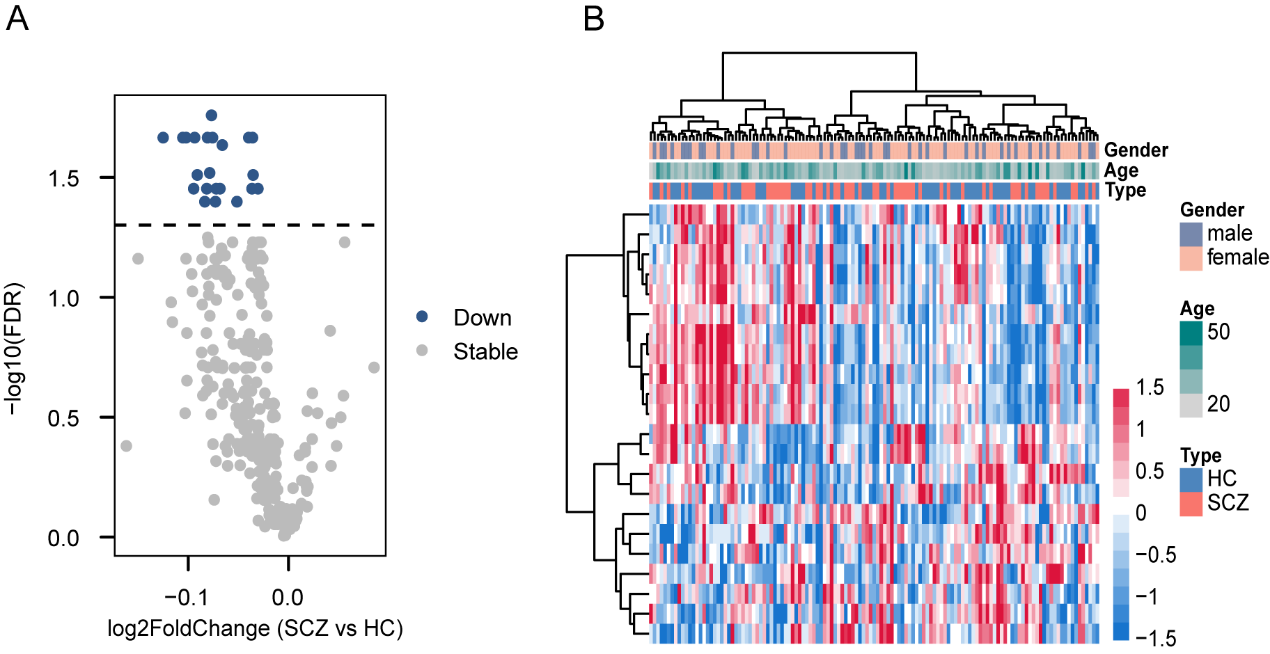


**Figure S4. Differential brain regions between individuals with schizophrenia (SCZ) and healthy controls (HCs).** **A**, Volcano plot of significantly different brain regions between individuals with SCZ and HCs, which were selected by a threshold of adjusted *P* value < 0.05 (horizontal dotted line) and absolute log2(fold change)>0 (vertical dotted line). **B**, Heatmap of brain regions that significantly differed between the SCZ and HC cohorts.
